# Screening and Mapping for Head Blast Resistance in a Panel of CIMMYT and South Asian Bread Wheat Germplasm

**DOI:** 10.3389/fgene.2021.679162

**Published:** 2021-05-13

**Authors:** Xinyao He, Philomin Juliana, Muhammad R. Kabir, Krishna K. Roy, Rabiul Islam, Felix Marza, Gary Peterson, Gyanendra P. Singh, Aakash Chawade, Arun K. Joshi, Ravi P. Singh, Pawan K. Singh

**Affiliations:** ^1^International Maize and Wheat Improvement Center (CIMMYT), Mexico City, Mexico; ^2^Bangladesh Wheat and Maize Research Institute (BWMRI), Nashipur, Bangladesh; ^3^Instituto Nacional de Innovación Agropecuaria y Forestal (INIAF), La Paz, Bolivia; ^4^United States Department of Agriculture–Agricultural Research Service, Foreign Disease-Weed Science Research Unit, Fort Detrick, MD, United States; ^5^ICAR-Indian Institute of Wheat and Barley Research, Karnal, India; ^6^Department of Plant Breeding, Swedish University of Agricultural Sciences, Alnarp, Sweden; ^7^BISA/CIMMYT-India, New Delhi, India

**Keywords:** GWAS, resistance screening, *Magnaporthe oryzae*, *Pyricularia oryzae*, MoT

## Abstract

Wheat blast (WB) is a destructive disease in South America and its first outbreak in Bangladesh in 2016 posed a great risk to food security of South Asian countries. A genome wide association study (GWAS) was conducted on a diverse panel of 184 wheat genotypes from South Asia and CIMMYT. Phenotyping was conducted in eight field experiments in Bolivia and Bangladesh and a greenhouse experiment in the United States. Genotypic data included 11,401 SNP markers of the Illumina Infinium 15K BeadChip and four additional STS markers on the 2NS/2AS translocation region. Accessions with stable WB resistance across experiments were identified, which were all 2NS carriers. Nevertheless, a dozen moderately resistant 2AS lines were identified, exhibiting big variation among experiments. Significant marker-trait associations (MTA) were detected on chromosomes 1BS, 2AS, 6BS, and 7BL; but only MTAs on 2AS at the 2NS/2AS translocation region were consistently significant across experiments. The resistant accessions identified in this study could be used in production in South Asian countries as a preemptive strategy to prevent WB outbreak.

## Introduction

Bread wheat grown in tropical and subtropical regions is subjected to a range of diseases, among which is wheat blast (WB). This is an emerging disease with growing impacts on wheat production in South America, South Asia, and Africa, and is a potential threat to other major wheat producers like India, United States and China (Duveiller et al., 2016; Cruz and Valent, 2017; Tembo et al., 2020). The disease had been endemic in four South American countries, Brazil, Bolivia, Paraguay, and Argentina, before its outbreak in Bangladesh in 2016, affecting 15,000 ha wheat fields with yield losses of 5–51% (Islam et al., 2016; Malaker et al., 2016). Soon thereafter, this disease was identified in the Muchinga Province of Zambia during the 2017–2018 rainy season, indicating its introduction into the African continent (Tembo et al., 2020).

The causal agent of WB is the *Triticum* pathotype of the ascomycete fungus *Magnaporthe oryzae* (MoT), which is closely related to the notorious pathogen of rice blast, the *Oryzae* pathotype of *M. oryzae*, but cross infection normally does not happen (Gladieux et al., 2018). Spike infection is the most conspicuous symptom of this disease, showing bleached spikelets above the infection point on rachis, which, together with the often-appeared gray mold on the infected rachis, are typical diagnostic symptoms of WB. Besides spike, MoT can infect all the above ground parts of a wheat plant, especially leaves, where the lesions are elliptical with a white center and a brown margin on the upper side and grayish mold on the underside (Igarashi, 1990). Leaf infection is less prominent than spike infection and usually does not lead to severe yield loss; but the fungal conidia produced on leaves could be an important source of inoculum for spike infection (Cruz et al., 2015).

The disease is favored by hot and humid conditions around anthesis, and according to Cardoso et al. (2008), the optimal temperature for the infection was at 30°C with extended wetting period. WB develops very fast under conducive conditions, and a severe infection might be developed only a few days after the first appearance of WB symptom, which gives no time for fungicidal application (Duveiller et al., 2016). Chemical control has major drawbacks in WB management, including low control efficiencies and fungal resistance (Castroagudín et al., 2015; Cruz et al., 2018). Under such circumstances, varietal resistance to WB is indispensable, as well as being economical and environment friendly. Field resistance of wheat against WB is a quantitative trait, and no immunity has been identified (Cruz et al., 2016). Several varieties have shown moderate disease resistance or tolerance in WB screening experiments or in long-term production in WB epidemic regions, such as BR 18 and CD 116 in Brazil, Montacu and Urubo in Bolivia, and Caninde#1 and Itapua 75 in Paraguay (Kohli et al., 2011). However, it was later found that many such varieties are 2NS/2AS translocation carriers, indicating a very narrow genetic variation (Cruz et al., 2016).

The 2NS chromosomal segment was initially introduced from *Aegilops ventricosa* (Zhuk.) into wheat genome to utilize rust resistance genes *Lr37*, *Sr38*, and *Yr17* (Helguera et al., 2003). Later, additional resistance genes have been identified from this chromosomal segment, like *Cre5* for cereal cyst nematode resistance (Jahier et al., 2001), *Rkn3* for root-knot nematodes resistance (Williamson et al., 2013), as well as WB resistance mentioned above, contributing to 64-81% reduction in head blast severity in Cruz et al. (2016). Recently, Juliana et al. (2019) reported a prominent role of the 2NS/2AS translocation in CIMMYT germplasm, conferring yield advantage, lodging resistance, along with blast and rust resistance. Apart from the 2NS/2AS translocation, there have been very limited blast resistance genes identified for MoT, i.e., *Rmg2*, *Rmg3*, *Rmg7*, *Rmg8*, *RmgTd(t)*, and *RmgGR119* (Zhan et al., 2008; Cumagun et al., 2014; Tagle et al., 2015; Cruz et al., 2016; Wang et al., 2018a). However, most of these genes have been overcome by new MoT isolates and only *Rmg8* and *RmgGR119* remained to be validated in field experiments with new MoT isolates.

Since the introduction of MoT from South America to Bangladesh, the affected areas have been increasing, despite the unfavorable weather conditions witnessed in the last few years (Islam et al., 2019), demonstrating the adaptation of the pathogen to South Asian environment. This poses a great risk to the neighboring countries, especially India that shares a very long international border with Bangladesh and relies heavily on wheat production for food security (Mottaleb et al., 2019). Being both air- and seed-borne, MoT could be easily blown by wind or introduced via trade to the neighboring countries. Therefore, it is imperative to screen South Asian wheat germplasm for WB resistance with the hope to identify resistant genotypes that could be used in production/breeding for WB resistance. It will help to prepare for the scenario if WB occurs and spreads in these countries. The objectives of the current study were designed with this aim and includes (1) screen a panel of CIMMYT and South Asian bread wheat germplasm for adult plant WB resistance and (2) identify molecular markers associated with WB resistance via genome-wide association study (GWAS).

## Materials and Methods

### Plant Materials

A panel of 184 spring wheat genotypes was used in the present study. The genotypes were obtained from CIMMYT-Mexico (97 genotypes), India (40), Bangladesh (19), and Nepal (28). In the last three groups, there are genotypes directly introduced from CIMMYT due to extensive traditional utilization of CIMMYT germplasm in these countries.

### Field Experiments

The field experiments took place in three locations, Quirusillas and Okinawa in the Department of Santa Cruz, Bolivia, and Jashore in the Division of Khulna, Bangladesh. Field trials were performed in the 2017–2018 and 2018–2019 cropping seasons in Quirusillas, the 2018 cropping season in Okinawa, and the 2017-18 cropping season in Jashore. Two sowings of approx. 14 days apart were adopted in each trial, totally making eight experiments, which were named as per the location (“Quir” for Quirusillas, “Oki” for Okinawa, and “Jash” for Jashore), cropping season (“18” for the 2017–2018 or 2018 cycle, and “19” for the 2018–2019 cycle), and sowing (“a” for the first sowing and “b” for the second). For example, Quir18b represents the second sown experiment in the 2017–2018 cycle conducted in Quirusillas.

The cropping cycle runs from December to April in Quirusillas and Jashore, and from May to September in Okinawa. Field experimental units were 1-m double rows separated by 20-cm spacing in all three locations, and no replication was set within each sowing. A field misting system was equipped at each site to create a micro-environment favorable for WB infection. The system worked from 8am to 7pm in Quirusillas and Okinawa and from 9am to 5pm in Jashore, with a 10-min-misting per hour, during the WB development period. Field inoculation was done twice in all the experiments, with the first at anthesis and the second at two days after anthesis, using a backpack sprayer. Inoculum was a mixture of locally collected MoT isolates with high pathogenesis, which included isolates QUI1505, QUI1601, QUI1612, OKI1503, and OKI1704 in Quirusillas and Okinawa, and BHO17001, MEH17003, GOP17001.2, RAJ17001, CHU16001.3, and JES16001 in Jashore. The first three letters of the isolate names indicate the place of collection, followed by two digits for the year of collection and the rest digits as isolate identifiers. The MoT isolates were cultured on oatmeal agar medium following the protocol by He et al. (2020b), and the inoculum was adjusted at a concentration of 80,000 spores/mL before field application. Two local checks were used in each experiment, which are Urubo (resistant check) and Atlax (susceptible check) in Bolivia and BARI Gom 33 (resistant check) and BARI Gom 26 (susceptible check) in Bangladesh.

WB evaluation was performed on 10 spikes that had been marked at anthesis at 21 days after the first inoculation, for which the total and infected number of spikelets were recorded. Incidence was derived from the proportion of spikes with blast infection and severity was calculated as the averaged percentage of infected spikelets. WB index was calculated with the formula WB index = incidence × severity, which was used in all subsequent analysis. In addition, days to heading (DH) and plant height (PH) were scored in all experiments.

### Greenhouse Experiment

In 2017, a greenhouse experiment was conducted in the biosafety level-3 laboratory at USDA-ARS, Foreign Disease-Weed Science Research Unit, Fort Detrick, MD, United States (nominated as US17 in this study). The accessions were sown into 15-cm-diameter pots containing a commercial potting medium (Metro-Mix 360; Hummert International, Earth City, MO, United States), which were arranged in an incomplete randomized block design with one replication. Wheat varieties Urubo and Glenn were used as resistant and susceptible checks, respectively. A MoT isolate B-71 collected in Okinawa (Bolivia) in 2012 was used. Inoculation production was carried out on homemade oatmeal agar according to Valent et al. (1991), and the spore concentration was adjusted to 20,000 spores/ml for inoculation. Wheat heads were inoculated approximately two days after full head emergence, with approximately 0.75 ml of inoculum applied for each accession using an airbrush (model 69492; Harbor Freight Tools, Camarillo, CA, United States). After inoculation, the heads were covered with black plastic bags (model S-12322BL; ULINE) moistened with water for 48 h to facilitate the infection. Percentage WB severity was evaluated at 12 to 14 days after inoculation, which was determined when the susceptible check Glenn exceeded 90% of WB severity.

### Genotyping

The panel was genotyped with Illumina Infinium 15K BeadChip at Trait Genetics GmbH, Germany. Markers with missing data points more than 10% or minor allele frequency less than 5% were excluded from further analysis. Four STS markers in the 2NS/2AS region were applied to evaluate their association with WB index and suitability for marker-assisted selection (MAS), including *Ventriup-LN2* reported by Helguera et al. (2003), *WGGB156* and *WGGB159* by Wang et al. (2018b), and *cslVrgal3* that was derived from a follow-up study of Seah et al. (2001) (E. Lagudah, pers. comm.).

### Linkage Disequilibrium and Kinship Analysis

The linkage disequilibrium (LD) parameter R^2^ among the markers was calculated using TASSEL 5^1^, and LD was visualized with R^2^ plotted against the physical distances. A kinship matrix and clusters among individual genotypes was generated with all the SNP markers.

### Statistical and GWAS Analysis

Analysis of variance (ANOVA) was performed with the PROC GLM module in SAS program ver. 9.2, and the results were used for calculating the heritability estimates, using the formula *H*^2^ = $\sigma_{g}^{2}$/($\sigma_{g}^{2}+\sigma_{g^{*}y}^{2}$/*y* + $\sigma_{g^{*}s}^{2}$/*s* +$\sigma_{e}^{2}$/*sy*) for experiments in Quirusillas and *H*^2^ = $\sigma_{g}^{2}$/($\sigma_{g}^{2}$ +$\sigma_{e}^{2}$/*s*) for those in Okinawa and Jashore, where $\sigma_{g}^{2}$ represents genetic variance, $\sigma_{g^{*}y}^{2}$ for genotype-by-year interaction,$\sigma_{g^{*}s}^{2}$ for genotype-by-sowing interaction, $\sigma_{e}^{2}$ for error variance, *y* for the number of years, and *s* for the number of sowing. Principal component analysis (PCA) on phenotypic data was conducted with the PAST software ver. 3.01 (Hammer et al., 2001).

Marker-trait association (MTA) tests were carried out in TASSEL 5 using the mixed linear model (MLM) (Yu and Buckler, 2006) with the optimum level of compression and the “population parameters previously determined” option. The first two principal components (PC) of the population structure were used as fixed effects, and the kinship among individuals estimated using the centered identity-by-state method (Endelman and Jannink, 2012) was used as a random effect in the mixed linear model. In addition, the multilocus mixed model (MLMM) and fixed and random model circulating probability unification (FarmCPU) models were also conducted using the R software package GAPIT v. 3.5 (Zhang et al., 2010). The *p*-values, additive effects and percentage variation explained by each marker were obtained and Manhattan plots with the −log10 *p*-values of the markers were plotted using the ‘R’ package CMplot. The Bonferroni method for multiple testing correction at an α level of 0.20 was used to declare significance of the markers.

## Results

### Phenotypic Evaluation

**FIGURE 1:**
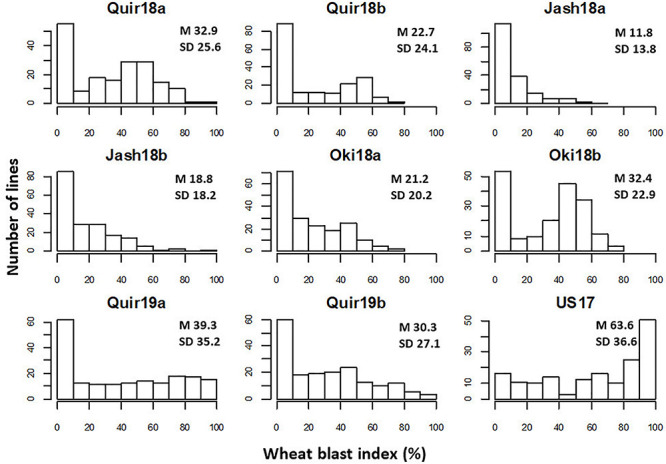
Histograms of wheat blast index in individual experiments. “Quir” stands for Quirusillas, “Jash” for Jashore, and “Oki” for Okinawa, “18” and “19” for the 2017-18 or 2018 cycle and 2018-19 cycle, respectively, and “a” and “b” for the first and second sowing, respectively. “US17” stands for the 2017 greenhouse evaluation in the United States. Grand mean (M) and standard deviation (SD) values are presented for all experiments.

**FIGURE 2:**
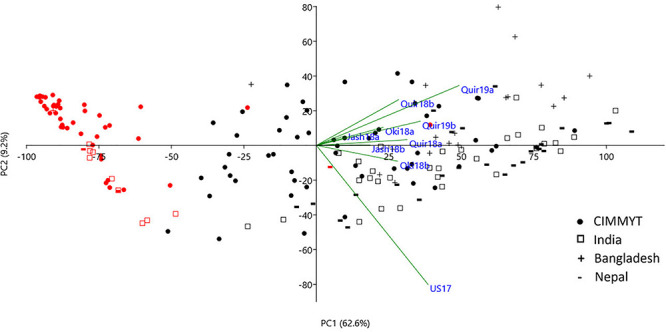
Principal component analysis (PCA) of the 184 accessions on wheat blast index across nice environments. Cosine of the angle between vectors indicates correlation between variables in the dimension of the first two principal components (PCs). *Red* symbols denote accessions with the 2NS/2AS translocation. Refer to Supplementary Figure 1 for a PCA chart with accession labels.

The WB disease pressure varied greatly among experiments, with Jash18a being the lowest with a grand mean of WB index of 11.8% and Quir19a the highest of 39.3% among the field experiments (Figure 1). The resistant and susceptible checks were among the lines of the lowest and the highest infection, respectively, in all field trials (Supplementary Table 1). The greenhouse experiment US17, however, exhibited a much higher disease pressure, showing a grand mean of WB severity of 63.6%. A bimodal distribution of the genotypes was observed in most of the experiments, except for the ones with lower disease pressure, like Jash18a and Jash18b (Figure 1). ANOVA indicated significant effects of “Genotype” in all three locations, as well as the significant effects of “Year” in Quirusillas and “Sowing” in Okinawa and Jashore. A high heritability estimate of 0.84 was obtained for Quirusillas; but only moderate heritability estimates from 0.66 to 0.69 were obtained for the other two locations (Table 1). Phenotypic correlations of WB among experiments were all significant, with *r* values ranging from 0.27 to 0.72. Among the experiments, Jash18a that exhibited the lowest disease pressure showed generally poor correlation with others, whereas other experiments, especially those in Bolivia, exhibited better correlations (Table 2). According to the PCA results, however, it was US17 that had the least association with the rest experiments at the dimension of the first two PCs (Figure 2).

**TABLE 1:** Analysis of variance for wheat blast index in different locations and its heritability estimates.

**Location**	**Source**	**DF**	**Mean square**	***F* value**	***P* value**	**Heritability**
Quirusillas	Genotype	183	2273.20	6.18	<0.0001	0.84
	Year	1	9247.88	25.16	<0.0001	
	Sowing (Year)	1	41.40	0.11	0.7375	
	Genotype × Year	183	333.07	0.91	0.7469	
	Genotype × Sowing	183	226.06	0.62	0.9995	
	Error	182	367.56			
Jashore	Genotype	183	387.20	2.92	<0.0001	0.66
	Sowing	1	4453.70	34.39	<0.0001	
	Error	183	132.42			
Okinawa	Genotype	183	715.00	3.26	<0.0001	0.69
	Sowing	1	11663.17	53.23	<0.0001	
	Error	182	219.12			

**TABLE 2:** Pearson correlation coefficients of wheat blast index among the 9 environments.

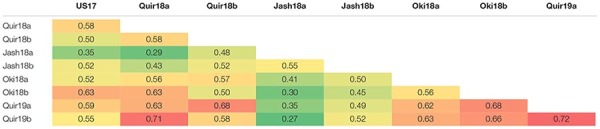

*All correlations were significant at *P* < 0.0001. “Quir” stands for Quirusillas, “Jash” for Jashore, and “Oki” for Okinawa, “18” and “19” for the 2017–2018 or 2018 cycle and 2018–2019 cycle, respectively, and “a” and “b” for the first and second sowing, respectively. “US17” stands for the 2017 greenhouse evaluation in the United States. Cell shades change from green to red with the increase of correlation coefficients.*

In both Jashore and Okinawa, the second sown experiments got higher infection than the first sown ones, whereas a reverse trend was found in Quirusillas (Figure 1). However, within a same sowing, no significant correlation was found between WB and DH except for Jash18b, with a low *r* value of 0.17. Similarly, significant correlation between WB and PH was only found in Quir19a and Quir19b, with low *r* values of around −0.20 (Table 3).

**TABLE 3:** Phenotypic correlation of field wheat blast index with days to heading (DH) and plant height (PH) in individual experiments.

	**Quir18a**	**Quir18b**	**Jash18a**	**Jash18b**	**Oki18a**	**Oki18b**	**Quir19a**	**Quir19b**
**DH**	−0.06	−0.13	0.07	0.17*	−0.06	0.06	−0.01	−0.04
**PH**	−0.13	−0.11	0.13	0.01	−0.07	−0.04	−0.24**	−0.18**

“Quir” stands for Quirusillas, “Jash” for Jashore, and “Oki” for Okinawa, “18” and “19” for the 2017–2018 or 2018 cycle and 2018–2019 cycle, respectively, and “a” and “b” for the first and second sowing, respectively. *p < 0.05; ** *p* < 0.01.

### Genotyping, Population Structure and Linkage Disequilibrium

This part of results has been reported in our previous study on tan spot resistance of the same panel (Phuke et al., 2020), with the only difference being the incorporation of the four 2NS-associated STS markers. Briefly, 11,405 markers of good quality were selected after filtering for the subsequent analysis, with generally good genomic coverage for A- and B-genome chromosomes but not for D-genome chromosomes (Supplementary Figure 2). Two subpopulations were identified in both Kinship and PCA analysis, of which the small sub-population comprised only nine Indian accessions (Supplementary Figures 3, 4). The big sub-population could be further divided into two groups, of which the small group is composed of only South Asian lines, whereas the big group comprises CIMMYT lines and some South Asian accessions with CIMMYT parentage (Supplementary Figure 4). The average extent of LD was estimated at 25 Mb, a physical distance over which R^2^ decayed to a critical value of 0.10 across the genome.

### GWAS Results

**FIGURE 3:**
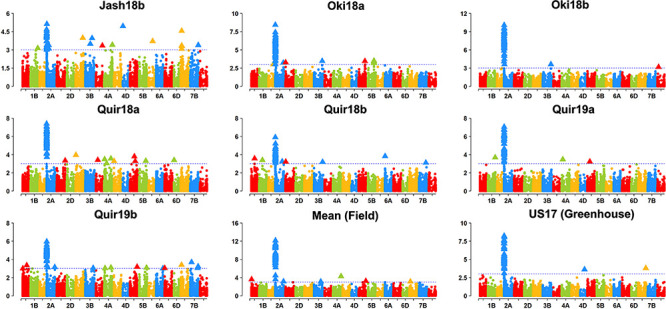
Manhattan plots based on MLM model. “Quir” stands for Quirusillas, “Jash” for Jashore, and “Oki” for Okinawa, “18” and “19” for the 2017-18 or 2018 cycle and 2018-19 cycle, respectively, and “a” and “b” for the first and second sowing, respectively. “US17” stands for the 2017 greenhouse evaluation in the United States. Jash18a did not fit this model and is not shown, instead a plot for mean data across the rest seven field trials is presented.

Based on the Bonferroni method, *p* = 1.74E-5 was determined as the significant level of MTAs. However, only markers on the 2NS/2AS translocation regions exceeded this threshold regardless of the GWAS algorithm used. When the threshold of *p* was reduced to 0.001, some significant SNPs on other chromosomes were detected, but none of them could be repeatedly detected across experiments (Figure 3). With MLMM and FarmCPU, only three markers outside the 2NS/2AS region could be detected in two of the nine experiments, being located on 1BS, 6BS, and 7BL, with their *p* values mostly higher than 1.74E-5 and could only be regarded as putative MTA loci (Table 4). It is noteworthy that the model MLM did not fit Jash18a; but MLMM and FarmCPU that fitted this experiment were not able to identify a stably expressed MTA locus either. In order to detect potential MTAs that might have been masked by the strong phenotypic effects of 2NS, the non-2NS lines were analyzed independently for GWAS; nevertheless, no repeatable MTA could be found (data not shown).

**TABLE 4:** Markers significantly associated with wheat blast resistance.

**Algorithm**	**SNP**	**Chromosome**	**Position (Mb)**	***P* value**	***R*^2^**	**Experiment**
MLM, MLMM, FarmCPU	Multiple SNPs	2NS/2AS	0-35.4	1.87E-13 to 9.35E-4	0.26-0.50	All
MLMM	IAAV2838	1BS	41.6	5.71E-4 to 8.95E-4	0.13	Oki18b, Quir19b
FarmCPU	AX-94523488	6BS	51.3	2.25E-4 to 2.63E-4	0.07	Oki18a, Quir19b
FarmCPU	AX-95215927	7BL	682.9	1.84E-7 to 1.26E-4	0.35	Quir18b, Quir19a

*Significant markers on 2NS/2AS are listed in Supplementary Table 1. Physical positions for SNPs are from Chinese Spring RefSeq ver. 1.0.*

### Effects of the 2NS/2AS Translocation on Blast Resistance

As the only major MTA locus identified in the current study, 2NS/2AS exhibited strong phenotypic effects on WB resistance. In the phenotype-based PCA analysis, PC1 mainly reflected WB resistance and separated most 2NS lines from the 2AS lines, clearly demonstrating the different WB resistance levels of the two groups (Figure 2). In total, 55 lines were identified as 2NS carriers, accounting for 29.9% of the entire panel. Majority of the 2NS lines were from CIMMYT, with only nine, three and one accession from India, Nepal and Bangladesh, respectively (Supplementary Table 1).

**FIGURE 4:**
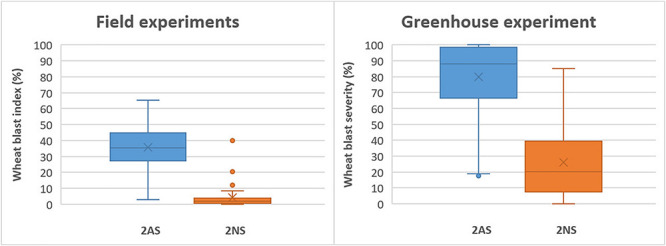
Phenotypic effects of the 2NS/2AS translocation on wheat blast resistance for field experiments in Bolivia and Bangladesh (left) and greenhouse experiment.

In the field experiments, the 2NS accessions exhibited a grand mean WB index of 3.8%, which was 35.7% for the 2AS lines. In the greenhouse experiment, however, the corresponding values became 26.0% and 79.6%, respectively, much higher than those in the field experiments (Figure 4). Despite the general trend, there were several 2NS carriers with higher WB infection, along with some 2AS carriers showing moderate resistance (Figures 2, 4). Unexpectedly, the famous 2NS donor, Milan, exhibited a grand mean of 39.9% in field experiments, probably due to the residual heterozygosity in the 2NS/2AS region (Supplementary Table 1).

As expected, the best performers in field experiments were mostly 2NS lines, whereas only four 2AS lines, CIM-5, CIM-11, CIM-32, and IND-29, exhibited a grand mean WB index less than 15%, a tentative threshold for WB resistance. However, in the greenhouse experiment, these four lines showed high levels of infection, with WB severities ranging from 66.6 to 100.0% (Supplementary Table 1), implying their vulnerability to WB under high disease pressure. Generally, the best 2NS lines performed more consistently across experiments, whereas the best 2AS lines were more subjective to environmental conditions, with wider ranges of WB index or severity (Supplementary Table 1).

## Discussion

Wheat blast had been an endemic disease of South America until its large-scale outbreak in Bangladesh in 2016 (Malaker et al., 2016), and its recent incidence in Zambia further corroborated the importance of preemptive actions against this disease in countries where WB is not detected (Tembo et al., 2020). Fortunately, WB has not been officially reported in any South Asian countries except Bangladesh, therefore, identification of WB resistant genotypes from the local wheat germplasm, as well as CIMMYT genotypes that have been extensively used in South Asian breeding programs, is an important finding to prevent or slow down the possible spread of WB in the region or worldwide.

In the current study, we evaluated 184 CIMMYT and South Asian wheat genotypes for their resistance to WB in eight field experiments and one greenhouse experiment. However, accessions with high and stable WB resistance were all 2NS carriers, in agreement with a previous study (Juliana et al., 2020). There were several 2AS lines showing resistance or moderate resistance under field conditions, though, they turned out to be susceptible in the greenhouse experiment. This implies that the currently identified non-2NS resistance is not as robust as the 2NS resistance under high disease pressure imposed under greenhouse conditions. Although misting systems were equipped for the screening nurseries in Bolivia and Bangladesh to maintain a conducive environmental condition for WB infection, greenhouse condition exerted a stronger disease pressure due to the constant humidity and temperature conditions favorable for WB infection (Cruppe et al., 2020). Several accessions of the current study have been evaluated under greenhouse condition in Bolivia and also exhibited higher WB severity (Marza et al., 2019). It is noteworthy that four 2AS lines, i.e., TEPOCA T89, QUAIU #1, SUPER 152, and SOKOLL/ROLF07, which Cruppe et al. (2020) reported as resistant or moderately resistant, were also included in the current study, but they exhibited wide ranges of WB across experiments, just like other promising 2AS lines (Supplementary Table 1). Nevertheless, we must say that such 2AS lines are still valuable in production, especially in terms of diversification of source of WB resistance. Because relying solely on 2NS is risky, due to the emergence of 2NS-virulent isolates that have already been found in South America (Ceresini et al., 2018; Cruppe et al., 2020). In production, these moderately resistant 2AS lines must be able to endure low to medium WB pressure; and under higher disease pressure, other management approaches especially fungicide application could be helpful in reducing yield loss (Cruz et al., 2018).

It is noteworthy that several accessions of the GWAS panel have been released in the WB affected or threatened countries. Two Indian accessions, HD 2967 (IND-1) and HD 3171 (IND-13) have been recommended to the farmers of WB prone areas in West Bengal that borders to Bangladesh^2^ (accessed in Jan. 30, 2021). A CIMMYT breeding line Kachu/Solala (BAW-1260, BGD-19) has been released in Bangladesh as BARI Gom 33 and in Bolivia as INIAF Okinawa, while another CIMMYT accession BORLAUG100 F2014 (CIM-35) has been released in Bangladesh as WMRI Gom 3, in Bolivia as INIAF Tropical, and in Nepal as Borlaug 2020. However, all these accessions are 2NS carriers, indicating the limited genetic variation in WB resistant sources, which needs to be complimented with non-2NS resistance. The implication for wheat breeding in the WB vulnerable regions is to combine different resistant sources to achieve a better and durable resistance, just like other wheat diseases (Singh et al., 2016). To achieve this goal, crosses have been made between the 2AS lines mentioned above and elite CIMMYT breeding lines with 2NS. Additional non-2NS resistant sources will be utilized in breeding once identified; but the 2NS resistance will still be a backbone for WB resistance breeding, considering its strong phenotypic effects.

The CIMMYT breeding line Milan (CM-34) is a WB resistance donor that has been widely used in South American, leading to many WB resistant varieties with Milan in their pedigree. Such varieties include Sausal CIAT that was released in Bolivia, CD 116 in Brazil, Caninde#1 in Paraguay, etc. (Kohli et al., 2011). However, according to our results, Milan might have some residual heterozygosity in the 2NS region, as evidenced by the many heterozygous genotypes of markers in the 2NS region (Supplementary Table 1). Indeed, upon genotyping multiple Milan plants with 2NS-associated markers, we did identify a few individuals that harbors 2AS instead of 2NS (data not shown). This is also reflected in the field experiments, where Milan plants with susceptible reaction to WB was often observed, although majority of the plants were resistant. Another explanation to this could be seed mixture, which is less possible since the Milan plants in the field appeared homogeneous apart from WB resistance. Therefore, marker diagnosis is needed to identify the 2NS-bearing Milan for crosses aimed at WB resistance.

Despite the attempts on different GWAS algorithms, MTAs on the 2NS/2AS region were the only stably expressed QTL across experiments, and MTAs in other chromosome regions were hardly repeatable and have not been reported in other genetic studies (Ferreira et al., 2020; Goddard et al., 2020; He et al., 2020b; Juliana et al., 2020). This could be caused by 1) genomic regions with major effects on WB resistance were not present in this panel, 2) they were of very low frequencies below the detection power of the GWAS algorithms employed in this study, or 3) bias introduced by the 15K SNP chip that was developed based on genotypes without WB resistance genes that otherwise would be detected in this study. Our previous study indicated an interval of about 2.3 Mb on 2NS that might harbor the underlying WB resistance gene (He et al., 2020b); however, in the present study, the significant markers on the 2AS/2NS region distributed evenly across the region, and no clear cluster with lower *p* values was identified, probably being caused by the lower mapping resolution. Therefore, larger GWAS panel or specifically designed fine mapping populations will be needed to better map the underlying gene. An additional observation was that STS markers *Ventriup-LN2* and *cslVrgal3* predicted the presence of 2NS better than *WGGB156* and *WGGB159* in Indian germplasm (Supplementary Table 1), which was unexpected since our previous results in a biparental population indicated that the latter two markers were closer to the WB resistance QTL (He et al., 2020b). A possible explanation to this could be a founder parent in Indian germplasm, in which the susceptible alleles of *WGGB156* and *WGGB159* were linked with the resistance allele of the WB QTL.

Phenological traits like PH and DH are often associated with disease resistance under field conditions, as frequently reported for Fusarium head blight (FHB, Xu et al., 2020), spot blotch (He et al., 2020a) etc. For field WB resistance, however, such association appeared to be less significant (He et al., 2020b). Based on the prevailing weather conditions, it is a general rule that early sowing in South America and late sowing in Bangladesh should be avoided to prevent severe WB infection (Cruz and Valent, 2017; He et al., 2020b). However, in a specific cropping cycle, this rule may not be true, e.g., the 2018 cycle in Okinawa, where the second sowing exhibited higher infection than the first (Figure 1). Similarly, the general trend for the association between DH and WB agreed mostly with that between sowing and WB; but the correlation coefficients were very low and mostly non-significant. Plant height mostly showed a negative correlation with WB index, possibly having a similar underlying mechanism to that in FHB, i.e. spikes of tall wheat plants are well ventilated to become drier compared to those of short plants (Yan et al., 2011), which is unfavorable for WB infection. Despite this, the correlations were mostly not significant, thus the impact of PH on WB infection must be marginal if not absent. Nevertheless, a GWAS or biparental population with low variation in DH and PH is always preferred in field experiments to reduce the influence from these confounding effects to have more consistent WB resistance results.

## Data Availability Statement

The data presented in the study are deposited in the Dataverse repository and can be accessed via the link https://hdl.handle.net/11529/10548559.

## Author Contributions

PS, RS, and AC conceived and designed the experiments. MK, KR, RI, FM, XH, and PS performed field trials. GP conducted the greenhouse experiments. GS, AJ, and RS provided germplasm. XH and PJ analyzed the data. XH wrote the first draft of the manuscript. All authors contributed and approved the final draft of the manuscript.

## Conflict of Interest

The authors declare that the research was conducted in the absence of any commercial or financial relationships that could be construed as a potential conflict of interest.

## Footnotes

1 http://www.maizegenetics.net

2 https://www.aicrpwheatbarley.org/wp-content/uploads/2020/07/Crop-Protection-Report-2019-20_compressed.pdf
