# Supplementary material for: Screening and Mapping for Head Blast Resistance in a Panel of CIMMYT and South Asian Bread Wheat Germplasm
Source: Front Genet. 2021 May 13;12:679162. doi: 10.3389/fgene.2021.679162 (PMC8155635; doi:10.3389/fgene.2021.679162)
Supplement: Supplementary file 2 [file Data_Sheet_1.docx]

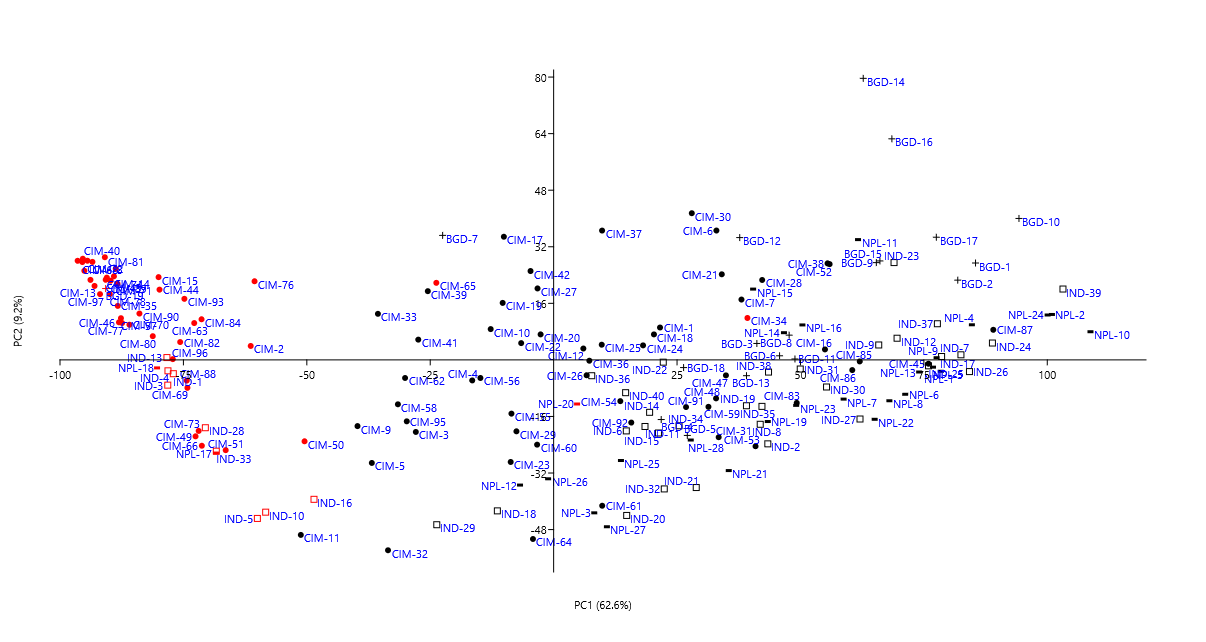

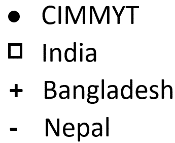


**Figure S1** Principal component analysis (PCA) of the 184 accessions on wheat blast index across nice environments. Red symbols denote accessions with the 2NS/2AS translocation.


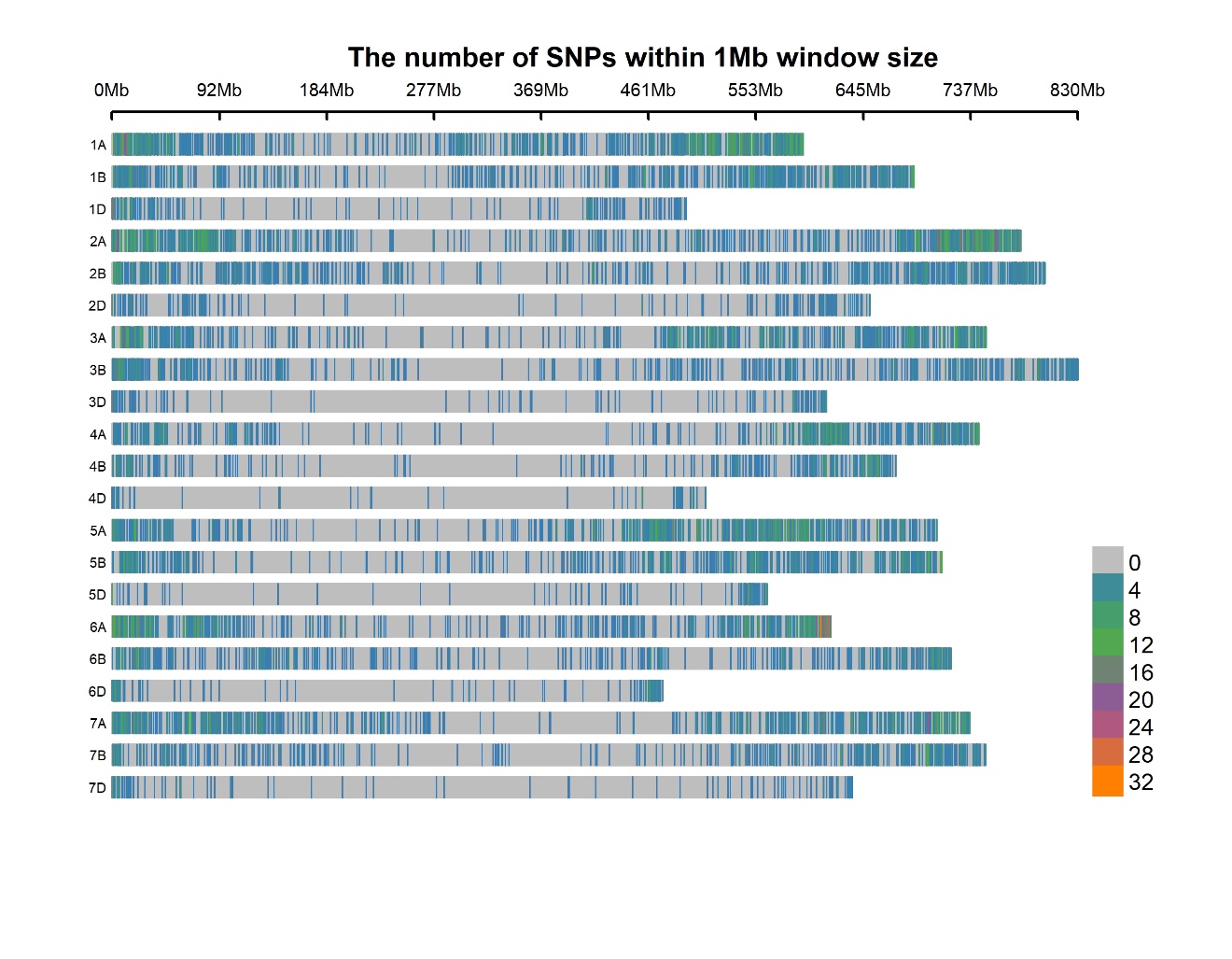


**Figure S2.** Genome-wide distribution of the molecular markers used in this study.


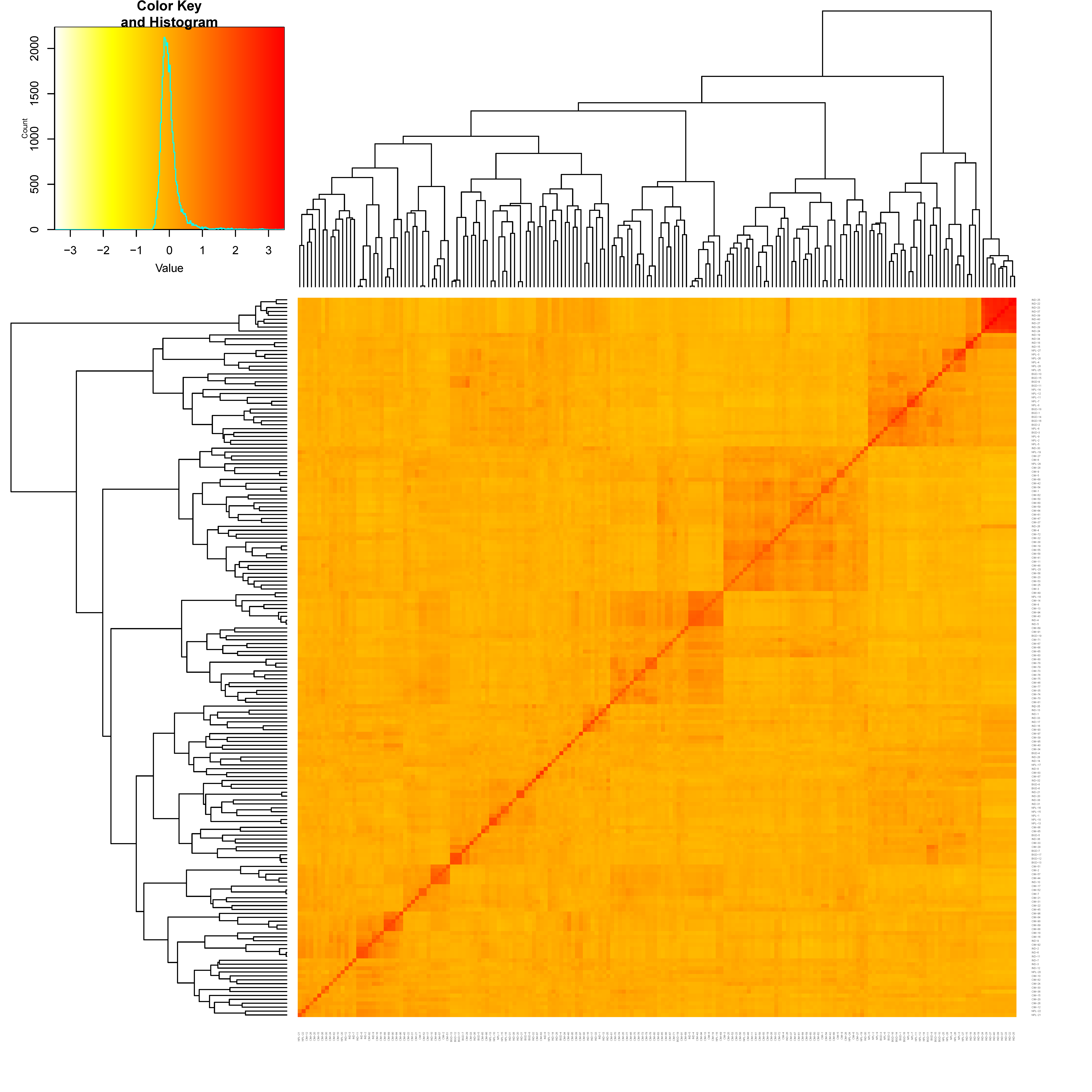


**Figure S3.** Heatmap and dendrogram of Kinship matrix estimated using Van Randen algorithm based on 11,405 molecular markers and 184 wheat genotypes.


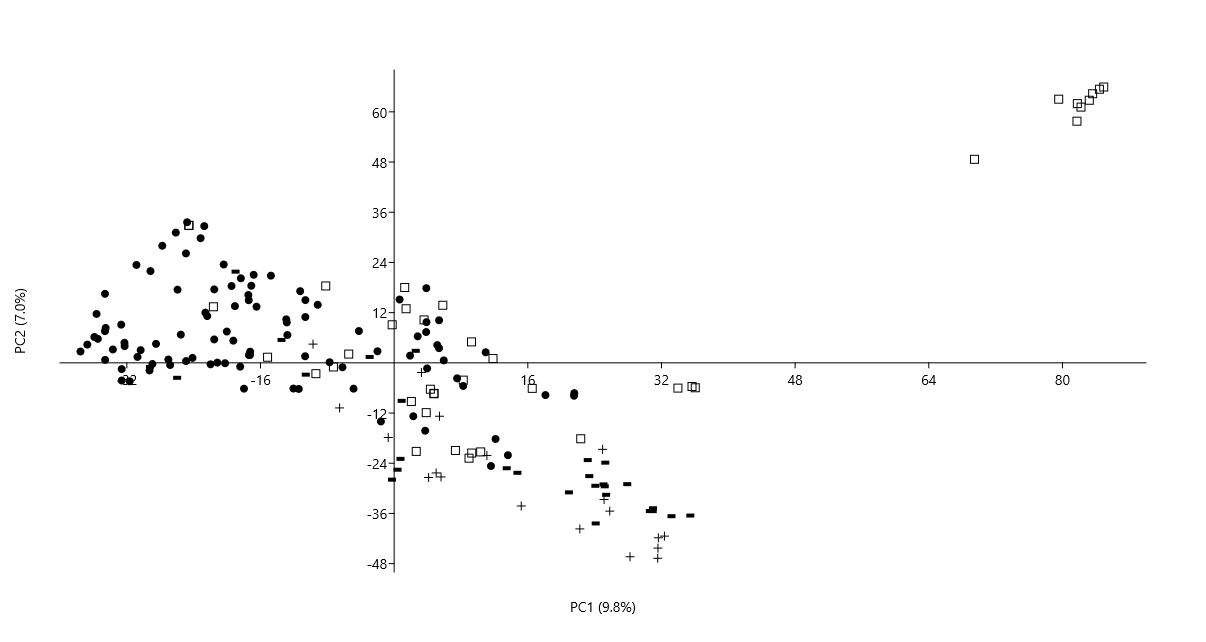

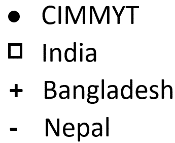


**Figure S4.** Principal component analysis (PCA) of the 184 accessions based on 11,405 molecular markers.
